# Domain Organization of Long Autotransporter Signal Sequences

**DOI:** 10.4137/bbi.s3411

**Published:** 2009-12-14

**Authors:** Jan A. Hiss, Gisbert Schneider

**Affiliations:** Johann Wolfgang Goethe-University, Chair for Chem- and Bioinformatics, Centre for Membrane Proteomics, Siesmayerstr. 70, D-60323 Frankfurt am Main, Germany. Email: hiss@bioinformatik.uni-frankfurt.de

**Keywords:** bacterial autotransporter, sequence analysis, pattern, protein targeting, signal peptide, protein trafficking

## Abstract

Bacterial autotransporters represent a diverse family of proteins that autonomously translocate across the inner membrane of Gram-negative bacteria *via* the Sec complex and across the outer bacterial membrane. They often possess exceptionally long N-terminal signal sequences. We analyzed 90 long signal sequences of bacterial autotransporters and members of the two-partner secretion pathway *in silico* and describe common domain organization found in 79 of these sequences. The domains are in agreement with previously published experimental data. Our algorithmic approach allows for the systematic identification of functionally different domains in long signal sequences.

## Introduction

Bacterial autotransporters translocate *via* the Sec complex across the inner membrane of Gram-negative bacteria and translocate themselves across the outer membrane.^1^,^2^ This is accomplished by a translocator domain at the C-terminus of the autotransporter which adopts a β-barrel fold within the outer membrane^3^ resembling a porin-like domain.^1^ The trimeric autotransporter consist of an N-terminal signal sequence, a central “passenger domain”, and a β-barrel forming translocation unit.^3^ The β-barrel domain is necessary for the secretion of the passenger domain and connected *via* an α-helical linker region.^4^ Bacterial autotransporters have been found in many Gram-negative bacteria and are often associated with virulence factors such as adhesion, biofilm formation, aggregation, invasion, and toxicity.^5^ For translocation across the inner bacterial membrane autotransporters possess N-terminal signal sequences.^2^ In 2007 Dautin and Bernstein reported around 10% of the know autotransporters to contain a signal sequence with more than 50 residues. These N-terminal signal sequences exhibit a tripartite organization (*n*, *h*, *c*) as described by von Heijne.^6^ According to this nomenclature, “n” refers to an N-terminal region of the signal peptide which varies in length and often contains charged residues. The “h” or core region is a hydrophobic stretch required for the interaction between the signal peptide and SRP.^7^ “c” refers to the signal peptidase cleavage site. Additionally they can be roughly divided into two domains: i) an N-terminal extension of about 25 residues, ii) a C-terminal part that resembles a signal peptide.^3^ This division is in two domains, where one is like a functional signal peptide and is strikingly similar to the “NtraC model” which has recently been introduced by the writers as a general model of long eukaryotic signal peptides.^8^

Henderson et al reported at least 80 proteobacterial autotransporters with a signal sequence of at least 40 residues and published a list containing 46 sequences.^1^ The authors propose four different regions based on hydrophobic and charged residue distribution (N1,H1,N2,H2) and a C region (cleavage site) following the standard *n, h, c* organization of export signals according to von Heijne.^6^ Desvaux et al continued this approach and termed the N2 and H2 region the “extended signal peptide region” (ESPR).^9^ They propose that the ESPR may be important for additional functions besides targeting. In this report, we extend and formalize this approach by proposing a dual domain organization proposed by our algorithm.

## Materials and Methods

We analyzed 16 autotransporters and two-partner secretion sequences published by Szabady et al^10^ and 35 further long signal sequences of bacterial autotransporters taken from Henderson et al.^1^ Two-partner secreted proteins are known to possess an N-terminal conserved region important for their secretion.^11^ Additionally we performed a sequence database search in UniProtKB/SwissProt Release 14.7^12^ using the sequence retrieval system (SRS, Release 7.1.3).^13^ We searched for proteobacterial sequences with an annotated similarity to an autotransporter domain and a signal sequence of at least 40 residues, resulting in 56 sequences. Of those 56 sequences 39 were not considered in the work of Henderson et al^1^ and Szabady et al.^10^ From the sequences considered suitable by the work of Henderson et al^1^ Szabady et al^10^ and our own database search we assembled a dataset of 90 sequences. The signal peptidase cleavage sites were used as suggested in Henderson et al^1^ Szabady et al^10^ and for the 39 sequences retrieved *via* SRS as annotated in SwissProt UniProtKB/SwissProt Release 14.7, respectively. The SwissProt database entries contain sequences with predicted or putative signal sequences.

The following sequences were omitted from our analysis due to minor sequence aberrations between the publications and the UniProtKB database entry: O32591, Q47692, Q54151 and Q8VSL2.

The following to YP_001161762 orthologous sequences were omitted since they possess an identical signal sequence: YP_001719317, YP_001874066, Q1C309, Q1CMJ2 and Q665P5. When two database entries contained the identical sequences one entry was omitted and both accession numbers are given.

In total, 90 signal sequences encompassing more than 40 residues from bacterial autotransporters were analyzed in this study and in regards to their possible internal domain organization.

The 28 long signal sequences not associated with autotransporters were retrivied from the UniProtKB/SwissProt Release 14.7^12^ using the Sequence Retrieval System (SRS, Release 7.1.3).^13^ We searched for “non-potential” bacterial signal sequences with evidence at protein level and a length of at least 40 residues. All retrived sequences contain the twinarginine (TAT)^14^ signal which leads to export to the periplasm or extracellular space (Suppl. Table 1).

The 228 short bacterial signal peptides associated with autotransporters were retrieved from the UniProtKB/SwissProt Release 14.7^12^ using SRS (Release 7.1.3).^13^ We searched for proteobacterial signal sequences with less than 40 residues that contain annotated similarities to known autotransporters (Suppl. Table 2).

The detection of the domains was performed using the NtraC algorithm,^8^ an algorithmic approach to identifying domains in long eukaryotic signal peptides based on secondary structure aspects. The NtraC model proposes one domain to be essential and sufficient for targeting while rendering the other domain free for additional functions. Here, “N” and “C” denote two potential domains: an N-terminal “N-domain” and a C-terminal “C-domain” predicted by the algorithm. The transition area between both domains is refered to as “tra”. The algorithm works on the complete signal peptide sequence and suggests the domain positions. The N- and C-domains contain targeting signals that are not detectable when the whole signal sequence is regarded as an entity as performed by current prediction software. Until recently six predicted domains have already been tested experimentally *in vitro*, from which five exhibit the predicted targeting function^8^ (Resch and Hiss in preparation).

## Results and Discussion

We analyzed 90 long signal sequences of bacterial autotransporters and the two-partner secretion pathway in regards to their potential two-domain (NtraC) organization.

Of the 16 signal sequences collected in Szabady et al^10^ 14 are predicted to have a two-domain organization (Table 1). Of the 46 autotransporter signal sequences collected in Henderson et al^1^ 35 are not listed in Szabady et al. Of those 35 sequences 32 are predicted to have a two-domain organization (Table 1). Of the 39 sequences we found via SRS and which are not described in Henderson et al^10^ or Szabady et al 2005, 31 are predicted to have a two-domain organization.

In total, from 90 long signal sequences considered in this study 77 (86%) are predicted, by our algorithm, to be organized in two domains.

For two additional sequences (Q2J0N4, CAR56027) an NtraC organization is predicted which in the context of this work could be regarded as a false-positive: No C-domain with a targeting capacity was detected. For Q2J0N4 an N-terminal mTP is predicted by TargetP^15^,^16^ and for CAR56027 a signal anchor by SignalP.^17^ If these two sequences are included a total of 79 of 90 (88%) signal sequences are predicted to be organized in two domains.

The two-domain organization proposed by the algorithm is in agreement with the ESPR of Desvaux et al^9^ and the conservation of the “N-terminal extension” reported by Szabady et al^10^ within a margin of ±5 residues.

Szabaday et al^10^ further reported a conserved sequence pattern in the N-terminal extension of autotransporter and two-partner secretion systems signal peptides.^10^ This conservation is also present in 43 of 46 sequences compiled by Henderson et al.^1^ For the long signals sequences extracted *via* SRS, the conserved pattern is only present in three sequences (Table 1, no. 52–54). One of these three (no. 54, Q8CWC7) is a Pic variant of a different *E.coli* strain (Table 1, Nr. 4). The remaining 36 sequences found *via* SRS do not show the conserved sequence motif reported by Szabady et al^10^ although they are annotated to contain a domain similar to autotransporters. This might argue for the sequences of Szabady et al^10^ to form a group. Nevertheless, we found that the SRS sequences have the same domain structure in their long signal peptide as the sequences reported by Szabady et al^10^ and Henderson et al.^1^ The N-terminal region of this group of autotransporters may have a different function not requiring this conserved motif pattern.

We want to highlight the case of the long signal peptide of EspP. EspP is an extracellular serine protease of *E. coli* which is divided into four subtypes α, β, γ and δ of which α and γ are proteolytically active.^18^,^19^ The long signal peptide of subtype EspPα contains the conserved sequence pattern reported by Szabady et al^10^ and for which experimental results were published by Peterson et al.^20^ These authors showed that residues 23–55 can act as an independent targeting signal. In 2006 Peterson proposed the N-terminal extension of the signal sequences to mediate an interaction with an unknown cytosolic factor or to induce an unusual signal peptide conformation prior to protein translocation.^21^ Notably, the analysis of the 55 residue signal sequence of EspP by our algorithm identified a two-domain (NtraC) organization:

– N-Domain (residues 1–26): unknown function,
– C-domain (residues 27–55): predicted secretion signal for Gram-negative bacteria.

The algorithm thereby proposed the same functional domain Peterson et al described experimentally.

We would like to stress that the NtraC algorithm is based on sequence information only and not influenced by the existing proposed fragmentation of long signal peptides. Our prediction method is therefore unbiased for the analysis of new sequences.

A further surprising result is the prediction of mitochondrial targeting peptides (mTP) for the proposed N-domains of the long bacterial signal peptides. In 17 of 90 (19%) cases the N-domain of a bacterial signal sequence is predicted as mTP (Table 1). Short bacterial signal peptides associated with autotransporters are in 29 of 228 (13%) cases predicted as mTP. As the presence of arginine is a typical feature for mTPs^15^,^16^ this could, in our case, lead to a prediction of a sequence as mTP if arginine residues are abundant. The positive charged residues are thought to form amphiphilic α-helices.^22^ This high abundance of positive charges (Table 2) has also been observed in the extended N-region of bacterial autotransporter signal sequences by Peterson et al.^21^ They reported a high net positive charge to be common in the N-terminus of serine protease autotransporters.

The automatic assignment “mTP” should thus not to be regarded as a perfect functional prediction but as the detection of a feature, namely the high abundance of charged residues. In 1994 Izard and Kendall reported that although a positive charge in the N-terminus may not be absolutely required for secretion^23^ a net negative charge or zero charge could result in considerably decreased rates of export.^24^–^26^ While Dierstein and Wickner reported that the N-terminal regions is not strictly required for processing by signal peptidase, 27 Peterson et al demonstrated that the positive charges in the N-terminal part of the bacterial signal sequences may influence SRP recognition.^20^

To investigate the role of charged residues *in silico* in the context of long signal sequences of autotransporters and their potential domain organization we counted the occurrence of charged residues in the N- and C-domain of all 79 autotransporter sequences predicted to be two-domain organized (Table 2). The border between the N-domain and the C-domain (transition area, “tra”) often contains charged residues. To take this into account the border between both domains was alternating, and included (+*tra*) or excluded (−*tra*) from the domains for the calculation (Table 2).

If the border between the domains was regarded as part of the C-domain(+*tra*), positively charged residues (His, Lys, Arg) occur approximately 1.6 times more often in the N-domain compared to the C-domain. Negatively charged residues (Asp, Glu) occur 2.3 times more often in the N-domain(−*tra*) compared to the C-domain(+*tra*). This difference becomes even more prominent if the border between both domains is counted as part of the N-domain(+*tra*) leading to 2.8 times higher occurrence of positively charged residues and 4.2 times higher occurrence of negatively charged residues in the N-domain compared to the C-domain.

This charge bias is an argument that charged residues may represent an inherent difference between the N- and the C-domain. The nearly three-fold increase in deviance between the N-(+*tra*) and C-domain(−*tra*) indicates that not only the presence of charged residues is of importance but also their position, favoring the N-terminal domain or between the two domains. The relative position curtly before the targeting signal in the C-domain could represent a characteristic feature.

The observed abundance of charged residues in the N-domain was also reported for the long signal peptides from vertebrata analyzed by us previously.^8^,^28^ The authors therefore propose that a potential additional function of the N-domain in long signal peptides is related to the abundance of positively charged residues in Gram-negative bacteria as well as in vertebrata. This is in agreement with the observation made by Peterson et al^20^,^21^ regarding a high net positive charge of the N-terminal part of the signal sequence and its potential influence on SRP recruitment. The NtraC algorithmic approach can be used to check individual observations, and pinpoint the sequence part that might be relevant for such an SRP interaction.

**Figure 1:**
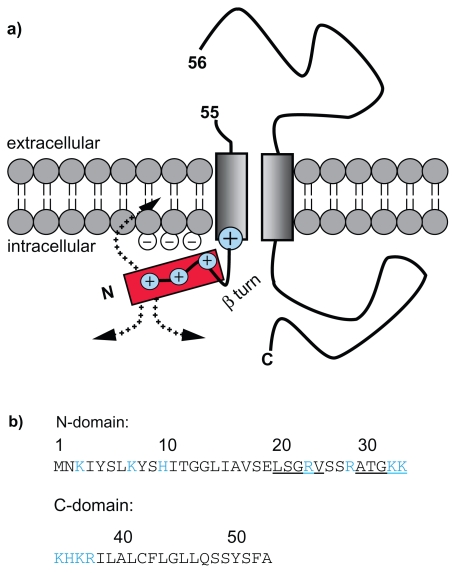
Cartoon of a possible membrane orientation of the signal peptide of EspP. **A**) Potential orientation of the N- and C-domain of the long signal peptide of EspP. **B**) N- and C-domain of the signal peptide of EspP. **Blue:** positively charged residues. **Underlined:** predicted β-turns.

**Figure 2:**
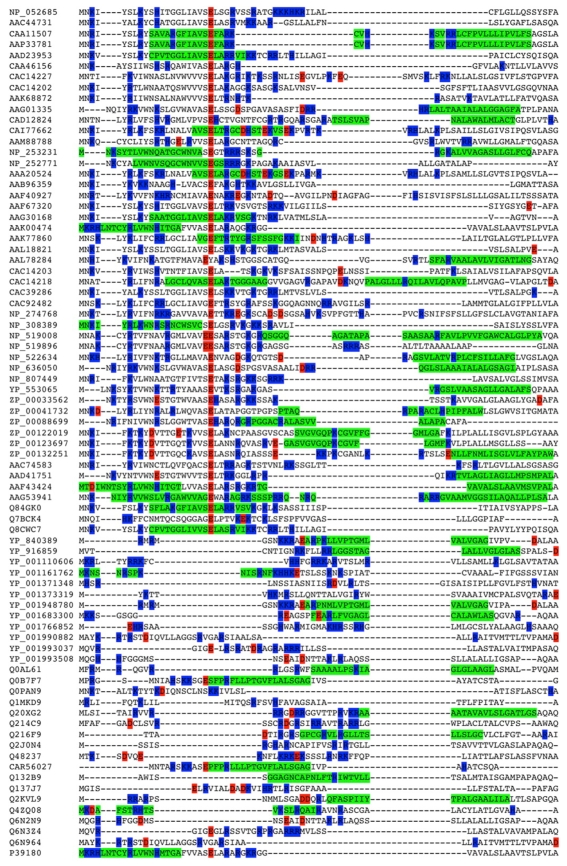
Multiple sequence alignment of 79 NtraC-organized autotransporter signal sequences (Matlab R2009a, Bioinformatics Toolbox Version 3.3). **Red:** negatively charged residues. **Blue:** positivly charged residues. **Green:** residues potentially part of an amphipatic helix.

A further hint towards a mechanistic aspect arises from to the secondary structure aspect of the NtraC model. As the C-domain of the signal peptide with its hydrophobic core is embedded in the membrane or the Sec complex during translocation, the N-domain may be kept in a defined angle to the membrane due to a predicted β-turn in the border between the N- and C-domain. The positive net charged of the N-domain could have the effect of keeping it outside and on top of the membrane. This might provide the means for the recruitment of other proteins (Fig. 1). We further report priliminary *in silico* results that 43 out of the 90 (48%) long signal peptide sequences of long autransporters and 21 out of 28 (75%) long bacterial signal peptides not associated with autotransporters could form an amphipathic helix. We compared this to short bacterial signal peptides associated with autotransporters and found that 34 out of 228 (15%) could form an amphipathic helix. The requirement to form an amphipathic helix was to possess nine adjacent amino acids in a helix in a window of 18 residues. In a second approach we allowed the adjacent nine e.g. polar residues to be interrupted by one e.g. non-polar residue and vice versa. Now 68 out of 90 (76%), 25 out of 28 (89%) and 58 out of 228 (25%) could form an amphipathic helix (Fig. 2). While one must keep in mind that short sequences in general provide less amino acids to form an amphipatic helix at all, we still report a tendancy of long singal peptides to form alpha helices.

## Conclusion

We present an extensive analysis of 90 long bacterial autotransporter signal sequences predicting in 86% of the sequences, a common two-domain organization. The described organization is in agreement with published experimental data and allows the identification of potential new domains *in silico* in long signal sequences. We corroborate the importance of charged residues in bacterial signal sequences and emphasize their position near the N-terminus as possible regularity. The approach highlights the relevance of charged residues in long signal sequences.

## Supplementary Materials

Table S1 28 long signal sequences not associated with autotransporters.

**Table:** 

>uniprot_features|AAUA_ALCFA_1 SIGNAL: Tat-type signal.
MRWLDKFGESLSRSVAHKTSRRSVLRSVGKLMVGSAFVLPVLPVARA
>uniprot_features|ABF2_STRCX_1 SIGNAL: Tat-type signal.
MCTREAVRMSREHDLPEIPSRRLLLKGAAAAGALTAVPGVAHA
>uniprot_features|CHOD_BREST_1 SIGNAL: Tat-type signal.
MTDSRANRADATRGVASVSRRRFLAGAGLTAGAIALSSMSTSASA
>uniprot_features|CHOD_STRS0_1 SIGNAL: Tat-type signal.
MTAQQHLSRRRMLGMAAFGAAALAGGTTIAAPRAAAAAKSAA
>uniprot_features|CPRA_DESHA_1 SIGNAL: Tat-type signal.
MENNQKRQQSGMSRRSFLKVGAAATTMGVIGAIKAPAKVANA
>uniprot_features|DHAQ_ACEPO_1 SIGNAL: Tat-type signal.
MGRLNRFRLGKDGRREQASLSRRGFLVTSLGAGVMFGFARPSSA
>uniprot_features|DHML_METEX_1 SIGNAL: Tat-type signal.
MLGKSQFDDLFEKMSRKVAGHTSRRGFIGRVGTAVAGVALVPLLPVDRRGRVSRANA
>uniprot_features|DHML_PARDE_1 SIGNAL: Tat-type signal
MLGNFRFDDMVEKLSRRVAGQTSRRSVIGKLGTAMLGIGLVPLLPVDRRGRVSRANA
>uniprot_features|DHML_PARVE_1 SIGNAL: Tat-type signal.
MLGNFRFDDMVEKLSRRVAGRTSRRGAIGRLGTVLAGAALVPLLPVDRRGRVSRANA
>uniprot_features|DMSA_ECOLI_1 SIGNAL: Tat-type signal.
MKTKIPDAVLAAEVSRRGLVKTTAIGGLAMASSALTLPFSRIAHA
>uniprot_features|DMSA_RHOCA_1 SIGNAL: Tat-type signal.
MTKFSGNELRAELYRRAFLSYSVAPGALGMFGRSLLAKGARA
>uniprot_features|DMSA_RHOSH_1 SIGNAL: Tat-type signal.
MTKLSGQELHAELSRRAFLSYTAAVGALGLCGTSLLAQGARA
>uniprot_features|GADH3_PECCY_1 SIGNAL: Tat-type signal.
MSEHKNGHTRRDFLLRTITLAPAMAVGSTAMGALVAPMAAGA
>uniprot_features|GFO_ZYMMO_1 SIGNAL: Tat-type signal.
MTNKISSSDNLSNAVSATDDNASRTPNLTRRALVGGGVGLAAAGALASGLQA
>uniprot_features|MBHS_AZOVI_1 SIGNAL: Tat-type signal.
MSRLETFYDVMRRQGITRRSFLKYCSLTAAALGLGPAFAPRIAHA
>uniprot_features|MBHS_ECOLI_1 SIGNAL: Tat-type signal.
MNNEETFYQAMRRQGVTRRSFLKYCSLAATSLGLGAGMAPKIAWA
>uniprot_features|MBHS_OLICO_1 SIGNAL: Tat-type signal.
MTPTETFYEVMRRQGVTRRSFLKFCSLTATALGLGPAYTSEIAHA
>uniprot_features|MBHS_RALEH_1 SIGNAL: Tat-type signal.
MVETFYEVMRRQGISRRSFLKYCSLTATSLGLGPSFLPQIAHA
>uniprot_features|NOSZ_ACHCY_1 SIGNAL: Tat-type signal.
MESKEHKGLSRRALFSATAGSAILAGTVGPAALSLGAAGLATPARA
>uniprot_features|NOSZ_PARDE_1 SIGNAL: Tat-type signal.
MESKQEKGLSRRALLGATAGGAAVAGAFGGRLALGPAALGLGTAGVATVAGSGAALA
>uniprot_features|NOSZ_PSEST_1 SIGNAL: Tat-type signal
MSDKDSKNTPQVPEKLGLSRRGFLGASAVTGAAVAATALGGAVMTRESWAQA
>uniprot_features|PHNS1_DESVH_1 SIGNAL: Tat-type signal
MRFSVGLGKEGAEERLARRGVSRRDFLKFCTAIAVTMGMGPAFAPEVAR
>uniprot_features|PHNS_DESFR_1 SIGNAL: Tat-type signal.
MNFSVGLGRDDAEKRLVQNGVSRRDFMKFCATVAAAMGMGPAFAPKVAE
>uniprot_features|PHNS_DESVM_1 SIGNAL: Tat-type signal.
MKISIGLGKEGVEERLAERGVSRRDFLKFCTAIAVTMGMGPAFAPEVARA
>uniprot_features|XYNC_STRLI_1 SIGNAL: Tat-type signal.
MQQDGTQQDRIKQSPAPLNGMSRRGFLGGAGTLALATASGLLLPGTAHA
>uniprot_features|YAGT_ECOLI_1 SIGNAL: Tat-type signal
MSNQGEYPEDNRVGKHEPHDLSLTRRDLIKVSAATAATAVVYPHSTLAASVPA
>uniprot_features|YEDY_ECOLI_1 SIGNAL: Tat-type signal
MKKNQFLKESDVTAESVFFMKRRQVLKALGISATALSLPHAAHA
>uniprot_features|YNFE_ECOLI_1 SIGNAL: Tat-type signal
MSKNERMVGISRRTLVKSTAIGSLALAAGGFSLPFTLRNAAAA

Table S2 228 short bacterial signal peptides associated with autransporters.

**Table:** 

>BIGA_SALTY_1 SIGNAL: Potential.
MNPMQKKKLISIAIALTLQSYYIPAIA
>ESTA_PSEAE_1 SIGNAL: Potential.
MIRMALKPLVAACLLASLSTAPQA
>ESTA_PSEPU_1 SIGNAL: Potential.
MIRMALKPLVAACLLASLSTAPQA
>HAP1_HAEIN_1 SIGNAL: Potential.
MKKTVFRLNFLTACVSLGIASQAWA
>HAP2_HAEIN_1 SIGNAL: Potential.
MKKTVFRLNFLTACISLGIVSQAWA
>IGA0_HAEIN_1 SIGNAL: Potential.
MLNKKFKLNFIALTVAYALTPYTEA
>IGA1_HAEIN_1 SIGNAL: Potential.
MLNKKFKLNFIALTVAYALTPYTEA
>IGA2_HAEIN_1 SIGNAL: Potential.
MLNKKFKLNFIALTVAYALTPYTEA
>IGA3_HAEIN_1 SIGNAL: Potential.
MLNKKFKLNFIALTVAYALTPYTEA
>IGA4_HAEIN_1 SIGNAL: Potential.
MLNKKFKLNFIALTVAYALTPYTEA
>IGA_NEIGO_1 SIGNAL:
MKAKRFKINAISLSIFLAYALTPYSEA
>LIP1_PHOLU_1 SIGNAL:
MKRSFIFAPGMLALSISAISNAHA
>OMPA_RICCN_1 SIGNAL: Potential.
MANISPKLFQKAIQQGLKAALFTTSTAAIMLSSSGALG
>OMPA_RICRI_1 SIGNAL: Potential.
MANISPKLFKKAIQQGLKAALFTTSTAA
>PERT_BORBR_1 SIGNAL:
MNMSLSRIVKAAPLRRTTLAMALGALGALGAAPAAHA
>PERT_BORPA_1 SIGNAL: Potential.
MNMSLSRIVKAAPLRRTTLAMALGALGAAPAAYA
>PERT_BORPE_1 SIGNAL:
MNMSLSRIVKAAPLRRTTLAMALGALGAAPAAHA
>PRTS_SERMA_1 SIGNAL:
MILNKRLKLAYCVFLGCYGLSIHSSLA
>PRTT_SERMA_1 SIGNAL: By similarity.
MILNKKLKLAYCVFLGCYGLSLHSSLA
>SCA1_RICCN_1 SIGNAL: Potential.
MNKLTEQHLLKKSRFLKYSLLASISVGA
>SCA1_RICFE_1 SIGNAL: Potential.
MNKLTEQNLLKKSRFLKYSLLASISVGA
>SCA2_RICCN_1 SIGNAL: Potential.
MNLQNSHSKKYVLTFFMSTCLLTSSFLSTSARA
>SCA2_RICFE_1 SIGNAL: Potential.
MSLQNSHSKKYVLTFFMSTCLLTSSFLSTSARA
>SCA2_RICSI_1 SIGNAL: Potential.
MSTCLLTSSFLSTSARA
>SSA1_PASHA_1 SIGNAL: Potential.
MYKIKHSFNKTLIAISISSFLSIA
>VACA1_HELPY_1 SIGNAL: Potential.
MEIQQTHRKINRPLVSLALVGALVSITPQQSHA
>VACA2_HELPY_1 SIGNAL:
MEIQQTHRKINRPLVSLALVGALVSITPQQSHA
>VACA3_HELPY_1 SIGNAL: Potential.
MEIQQTHRKINRPIISLALVGVLMGTELGA
>VACA4_HELPY_1 SIGNAL: Potential.
MEIQQTHRKINRPLVSLALVGALVSITPQQSHA
>VACA_HELPJ_1 SIGNAL: Potential.
MEIQQTHRKINRPLVSLVLAGALISAIPQESHA
>VACA_HELPY_1 SIGNAL: Potential.
MEIQQTHRKINRPLVSLALVGALVSITPQQSHA
>YADA1_YEREN_1 SIGNAL:
MTKDFKISVSAALISALFSSPYAFA
>YADA2_YEREN_1 SIGNAL:
MTKDFKISVSAALISALFSSPYAFA
>YADA_YERE8_1 SIGNAL: By similarity.
MTKDFKISVSAALISALFSSPYAFA
>YADA_YERPS_1 SIGNAL: By similarity.
MTKDFKISVSAALISALFSSPYAFA
>YAIT_ECOLI_1 SIGNAL: Potential.
MHSWKKKLVVSQLALACTLAITSQANA
>YAIT_SALTY_1 SIGNAL: Potential.
MHSWKKKLVVSQLALACTLAITSQANA
>YFAL_ECOLI_1 SIGNAL: Potential.
MRIIFLRKEYLSLLPSMIASLFS
>YPJA_ECOLI_1 SIGNAL: Potential.
MNRTSPYYCRRSVLSLLISALIYAPPGMA
>YTRP_PSEPU_1 SIGNAL: Potential.
MRKAPLLRFTLASLALACSQAFA
>YUAO_ECOLI_1 SIGNAL: Potential.
MCFFLGSRLAYA
>B0J5Y7_RHILT_1 SIGNAL: Potential.
MLRLTGLASTAALVLAVGPGWAQ
>B5ZHX8_GLUDA_1 SIGNAL: Potential.
MAGIFRLVLIASPFTAVTSVSFAQ
>B5ZKP4_GLUDA_1 SIGNAL: Potential.
MRVSVSSLAILCALRLALPHQASAQ
>O69257_BORPE_1 SIGNAL: Potential.
MNMSLSRIVKAAPLRRTTLAMALGALGAAPAAHA
>O69259_BORPE_1 SIGNAL: Potential.
MNMSLSRIVKAAPLRRTTLAMALGALGAAPAAHA
>O88143_BORPE_1 SIGNAL: Potential.
MNMSLSRIVKAAPLRRTTLAMALGALGAAPAAHA
>Q0P6P2_PSEPU_1 SIGNAL: Potential.
MRKAPLLRFTLASLALACSQALA
>Q1JYT2_DESAC_1 SIGNAL: Potential.
MTRYFLLAVLCVAILFAQPLQAS
>Q1K1B2_DESAC_1 SIGNAL: Potential.
MKKLYIIIPFLMMGLFPPAPCHAN
>Q1K3G2_DESAC_1 SIGNAL: Potential.
MRSLKMACPTLCVMLLTLCWSGLAAAY
>Q3R162_XYLFA_1 SIGNAL: Potential.
MQKIKNKFIVRTILATTVTTVLSACGG
>Q3R2M7_XYLFA_1 SIGNAL: Potential.
MERKNHKKTTLATLISVLLMGSAGATYAN
>Q3R323_XYLFA_1 SIGNAL: Potential.
MTSNFTRSLLAFAITLTTTQGIAK
>Q3R8A8_XYLFA_1 SIGNAL: Potential.
MERKNHKKTTLATLISVLLMGGAGATYAN
>Q3R9L6_XYLFA_1 SIGNAL: Potential.
MKLKFQKRKFLTVVIVFSMCGGSVVYAN
>Q3RA15_XYLFA_1 SIGNAL: Potential.
MTSNFTRSLLAFAITLTTTQGIAK
>Q3RAY9_XYLFA_1 SIGNAL: Potential.
MKTIFAARTILASALAAALSACGD
>Q3RB92_XYLFA_1 SIGNAL: Potential.
MKNMKTTFFPGSILVLTLVAFLSACGG
>Q3RE35_XYLFA_1 SIGNAL: Potential.
MQKIKNKFIVRTILATTVTTVLSACGG
>Q3REZ6_XYLFA_1 SIGNAL: Potential.
MKTIFAARTILASALAAALSACGD
>Q3RGD6_XYLFA_1 SIGNAL: Potential.
MTSNFTRSLLAFAITLTTTQGIAK
>Q3RGT2_XYLFA_1 SIGNAL: Potential.
MERKNHKKTTLATLISVLLMGGAGATYAN
>Q546U4_BORPE_1 SIGNAL: Potential.
MNMSLSRIVKAAPLRRTTLAMALGALGAAPAAHA
>Q5FY73_SALBN_1 SIGNAL: Potential.
MNKIYALKYSVRQGALVPV
>Q83YP8_ACTAC_1 SIGNAL: Potential.
MKILLKPFRYSVIATTIALVFNQPAFA
>Q9S3M8_BORPE_1 SIGNAL: Potential.
MNMSLSRIVKAAPLRRTTLAMALGALGAAPAAHA
>Q9S6M9_BORPE_1 SIGNAL: Potential.
MNMSLSRIVKAAPLRRTTLAMALGALGAAPAAHA
>A0B1Z1_BURCH_1 SIGNAL: Potential.
MFLALSGAGIVPAHATCSTAG
>A0LDC0_MAGSM_1 SIGNAL: Potential.
MKRSNLSLLTLSLATTGLLLMTQPAVAF
>A1JMW6_YERE8_1 SIGNAL: Potential.
MHKIYRFIKTFMVSCPVLLGGFSVVDA
>A1JSQ7_YERE8_1 SIGNAL: Potential.
MNINNIARLPCFRKTLLASLLVPLLTPLYSWA
>A4JIS0_BURVG_1 SIGNAL: Potential.
MNHRSFPLSRTRTGRRLAHSALIAGAVMPWASSAQ
>A4THP4_YERPP_1 SIGNAL: Potential.
MNNHKIWRLSAVAVALLISGNSYAD
>A4TMU3_YERPP_1 SIGNAL: Potential.
MKNSNTLNTRLLPLSILISSLVSGGAMAV
>A4TMU4_YERPP_1 SIGNAL: Potential.
MKSRHHLNTRLLPLSILISALIPAAVLAA
>A4TP40_YERPP_1 SIGNAL: Potential.
MDKTLLAGAISLSLVILPVQVLAF
>A4TQZ3_YERPP_1 SIGNAL: Potential.
MKTNRSTLSPCFRKTMIASLLVPLCSPLYSWAV
>A4W658_ENT38_1 SIGNAL: Potential.
MKTTFRLTQVATSISLLLGSSVVIPGTALAN
>A4W9S4_ENT38_1 SIGNAL: Potential.
MIGGVVSGFGILASPAALAA
>A4WEB5_ENT38_1 SIGNAL: Potential.
MKKKYLSQLISLLVASTAAQGLLTTHALAV
>A4WED8_ENT38_1 SIGNAL: Potential.
MRKLLKRSLLSQCVLMSLTSLSAFAA
>A4WGQ4_ENT38_1 SIGNAL: Potential.
MSKNITNPTAIDRRKVLGLSIGSAIALLSSAE
>A4XFF0_NOVAD_1 SIGNAL: Potential.
MKTNKSRLALGAASAAVAVGLAAQVQAA
>A4XXQ8_PSEMY_1 SIGNAL: Potential.
MALRVALIGLGLLQVATGVVAG
>A5FVP6_ACICJ_1 SIGNAL: Potential.
MKISRRFSLLAATALSLSACSG
>A5FX02_ACICJ_1 SIGNAL: Potential.
MGSEMTFETCVRRLAIGALAAGFIGLAPARAQ
>A5V6F0_SPHWW_1 SIGNAL: Potential.
MRYLLASTCLAAIAAVPVHAE
>A5VXL2_PSEP1_1 SIGNAL: Potential.
MRKAPLLRFTLASLALACSQALAG
>A5W1C9_PSEP1_1 SIGNAL: Potential.
MKSTSNPLRFDSIFYAVSTSLLLATPVETIAY
>A5W3S9_PSEP1_1 SIGNAL: Potential.
MRLRLMLTLGSLPLLGMVTPAQAN
>A5W741_PSEP1_1 SIGNAL: Potential.
MGIVKKQRGGPLVRAKVVMSALMMLSPIAQAL
>A6UG19_SINMW_1 SIGNAL: Potential.
MVVAKVRKCFSTVLSGALLAGLFCIVGSGEASAA
>A6X2Y0_OCHA4_1 SIGNAL: Potential.
MTGVLRHKSMLLMTTAALGFYVTTARGA
>A6X4K3_OCHA4_1 SIGNAL: Potential.
MHCRNIGSRAIRFLSSTALISLGTVLLQSTPGMAA
>A6X7J0_OCHA4_1 SIGNAL: Potential.
MKKLWLASTAIISASLFTSAAWSA
>A6X8H2_OCHA4_1 SIGNAL: Potential.
MLKRLNGKNVLFLRFLFLSAGTALAMTPVLAQ
>A7HZ72_PARL1_1 SIGNAL: Potential.
MTARNRTAAARRRHIAALMLGTALAALPHSGASAD
>A7IHM7_XANP2_1 SIGNAL: Potential.
MTVQSLFHGVSRTRALGLVAIGLGAGSLAQTVLAD
>A7IIP4_XANP2_1 SIGNAL: Potential.
MVGRSAVIRAAVLWTSIAAGTGAAHAQ
>A7IL68_XANP2_1 SIGNAL: Potential.
MPALYASSSRLVLSLFLVGMAAPALAA
>A8G7T5_SERP5_1 SIGNAL: Potential.
MPLKITRMPRPAVLAVAILCSMTTSALAY
>A8GG96_SERP5_1 SIGNAL: Potential.
MASFAPSFFSRGCLLALATTGGFSAVVNAA
>A8GHU3_SERP5_1 SIGNAL: Potential.
MKDKISHHLAVRKPLSKIYLALFSAPLLLMGSADMAR
>A8GIR8_SERP5_1 SIGNAL: Potential.
MKRLAIAIIAALPFCSAQAV
>A8GL40_SERP5_1 SIGNAL: Potential.
MKQATGKNKPALAPTWKLNALLCALLAAGGVQAA
>B0KJB0_PSEPG_1 SIGNAL: Potential.
MRKAPLLRFTLASLALACSQAFAA
>B0KMU3_PSEPG_1 SIGNAL: Potential.
MKSTSNPLRFDSIFYAVSTSLLLATPVETFAY
>B0KU28_PSEPG_1 SIGNAL: Potential.
MRCHRLLLPVALPLLTLPLLAHSQ
>B0KUR3_PSEPG_1 SIGNAL: Potential.
MIKRRNFTLSPLASAIGQLLLGASAVLFTGPAGAL
>B0KUS7_PSEPG_1 SIGNAL: Potential.
MHFTPNRLALCIALACAAFAPSAFAK
>B0T124_CAUSK_1 SIGNAL: Potential.
MQRKVLVATVATAPLLAMAFGAYAE
>B0T4N4_CAUSK_1 SIGNAL: Potential.
MMTRSSSKRTILAGSSLLVMAIAAAQPALAQ
>B0U200_XYLFM_1 SIGNAL: Potential.
MKNMKTTFFPGSILVLTLVAFLSACGG
>B0U225_XYLFM_1 SIGNAL: Potential.
MKTIFAARTILASALAAALSACGD
>B0U2J0_XYLFM_1 SIGNAL: Potential.
MQKIKNKFIVRTILATTVTTVLSACGG
>B0U5B1_XYLFM_1 SIGNAL: Potential.
MTSNFTRSLLAFAITLTTTQGIAK
>B0U5U7_XYLFM_1 SIGNAL: Potential.
MERKNHKKTTLATLISVLLMGGAGATYAN
>B0UED5_METS4_1 SIGNAL: Potential.
MRFLSGVSLAAVITAIMGVGAARAQ
>B1IVK2_ECOLC_1 SIGNAL: Potential.
MNRTSPYYCRRSVLSLLISALIYAPPGMAA
>B1IY80_ECOLC_1 SIGNAL: Potential.
MHQSGSVSLCRSAISVLVATALYSPIALAS
>B1J074_ECOLC_1 SIGNAL: Potential.
MHSWKKKLVVSQLALACTLAITSQANAA
>B1J4X2_PSEPW_1 SIGNAL: Potential.
MPSFSPVTLRHVLHVASLAPLLLLTPQAMAQ
>B1J6N8_PSEPW_1 SIGNAL: Potential.
MLQRLFCSLSLLTLAISAAHAA
>B1J6R7_PSEPW_1 SIGNAL: Potential.
MKSTSNPMRFDRIFYAVSTSMLLATPVETFAF
>B1JE37_PSEPW_1 SIGNAL: Potential.
MRKAPLLRFTLATLALACSQAFAA
>B1JGR2_YERPY_1 SIGNAL: Potential.
MDKTLLAGAISLSLVTLPVQVLAF
>B1JHT7_YERPY_1 SIGNAL: Potential.
MKKNRSTLSPCFRKTLIASLLVPLCSPLYSWAV
>B1JLC5_YERPY_1 SIGNAL: Potential.
MNNHKIWRLSAVAVALLISGNGYAD
>B1JPQ0_YERPY_1 SIGNAL: Potential.
MHNKFKANTLAISIAAILLSVSFNTLAV
>B1JSJ9_YERPY_1 SIGNAL: Potential.
MNTNSKKTYLSIAISSILYASTAMNANAD
>B1LTI2_METRJ_1 SIGNAL: Potential.
MTRGYGVSLAALAVALLGPGAQAQ
>B1YPU8_BURA4_1 SIGNAL: Potential.
MNRRRFPFSQARSGRRLAHSALIAGAVIPWPSAAQ
>B2FHR8_STRMK_1 SIGNAL: Potential.
MRMMQFTPKFPSAKNQSDLARAIATALLIATSGAAGA
>B2FI22_STRMK_1 SIGNAL: Potential.
MNHPLHGRSSHSRSPLHSRLALAVSSSLLLAAAAPAMA
>B2FPV5_STRMK_1 SIGNAL: Potential.
MERTMMVRSVLATALAMALTACG
>B2FSC8_STRMK_1 SIGNAL: Potential.
MLLSKRPIRTLMAAAIALAALPAMA
>B2FU91_STRMK_1 SIGNAL: Potential.
MKHSKLSLALAGLIAVGAIA
>B2FUQ4_STRMK_1 SIGNAL: Potential.
MYRAVPRAFRPRRLAVSVLQALAVPSLLLTTASVAWA
>B2I6G5_XYLF2_1 SIGNAL: Potential.
MKLKFQKRKFLTVVIVFSMYGGSVVYAN
>B2I753_XYLF2_1 SIGNAL: Potential.
MKNMKTAFFPGSILVLTLVAFLSACGG
>B2I7M9_XYLF2_1 SIGNAL: Potential.
MKTIFAARTILASALAAALSACGD
>B2I921_XYLF2_1 SIGNAL: Potential.
MERKNHKKTTLATLISVLLMGSAGATYAN
>B2I9C9_XYLF2_1 SIGNAL: Potential.
MTSNFTRSLLAFAITLTTTQGIAK
>B2JZU5_YERPB_1 SIGNAL: Potential.
MHNKFKANTLAISIAAILLSVSFNTLAV
>B2K394_YERPB_1 SIGNAL: Potential.
MNNHKIWRLSAVAVALLISGNGYAD
>B2K6L6_YERPB_1 SIGNAL: Potential.
MKKNRSTLSPCFRKTLIASLLVPLCSPLYSWAV
>B2K7Q9_YERPB_1 SIGNAL: Potential.
MDKTLLAGAISLSLVTLPVQVLAF
>B2K953_YERPB_1 SIGNAL: Potential.
MNTNSKKTYLSIAISSILYASTAMNANAD
>B2K9Q8_YERPB_1 SIGNAL: Potential.
MKSRSNLNTRLLPLSILISSLIPGAVLAA
>B3QEQ3_RHOPT_1 SIGNAL: Potential.
MKKQLLLTTSLAPLFAVGVLGGSPAHAD
>B3QI73_RHOPT_1 SIGNAL: Potential.
MVAGVVAIGLVGATSVSAQAQ
>B4EH84_BURCJ_1 SIGNAL: Potential.
MKRKARNLGLGGMAILTGAVPVSAYA
>B4EQ87_BURCJ_1 SIGNAL: Potential.
MGSNKKRAEARPKLLVPTGMLVALVGAGIVPA
>B4SIZ3_STRM5_1 SIGNAL: Potential.
MSRAVPRAFRPRRLAVSVLQALAVPSLLLTTAGVAWAG
>B4SJV7_STRM5_1 SIGNAL: Potential.
MNHPLYGRSSHSRSPRSRLALAVASCLLLAAATPATAS
>B4SLP2_STRM5_1 SIGNAL: Potential.
MHPFPALPAHRKAILGSALLAALLGMAAAPAARAS
>B4SLT8_STRM5_1 SIGNAL: Potential.
MRMMQFTPKFPSTLVRSRLGLAVASSLMLAAMGTANAV
>B4SP28_STRM5_1 SIGNAL: Potential.
MKHSKLSLALAGLLGIGAIMAA
>B4SRI9_STRM5_1 SIGNAL: Potential.
MERTTMVRSVLATALAMALTACGG
>B4STS4_STRM5_1 SIGNAL: Potential.
MQLSKHPIRSLMAAAIALAALPAMAG
>B5QTZ3_SALEP_1 SIGNAL: Potential.
MPTPQNYSFIAIAVSAALASMVFPSQA
>B5QUX9_SALEP_1 SIGNAL: Potential.
MTQKRTLLKYGILSLALAAPLSACA
>B5R2C8_SALEP_1 SIGNAL: Potential.
MNPMQKKKLISIAIALTLQSYYIPAIA
>B5R4M8_SALEP_1 SIGNAL: Potential.
MIVRKRRGRRTLCCLAGLMACSFFINTTYAWQ
>B5R4X4_SALEP_1 SIGNAL: Potential.
MHSWKKKLVVSQLALACTLAITSQANA
>B5R5W4_SALG2_1 SIGNAL: Potential.
MHSWKKKLVVSQLALACTLAITSQANA
>B5R6Z9_SALG2_1 SIGNAL: Potential.
MTQKRTLLKYGILSLALAAPLSACA
>B5RG61_SALG2_1 SIGNAL: Potential.
MPTPQNYSFIAIAVSAALASMVFPSQA
>B5RGN6_SALG2_1 SIGNAL: Potential.
MIVRKCRGRRTLCCLAGLMACSFFINTTYA
>B8ENQ5_METSB_1 SIGNAL: Potential.
MSDSNFNLKSLAAILLGGVALPLLLPASQALAA
>B8ILY6_METNO_1 SIGNAL: Potential.
MLGCESGRAHGLDRMRRLLLLGVSFAGLPVLATSALAQ
>O86135_BORPE_1 SIGNAL: Potential.
MHIYGNMNRATPCRGAVRALALALLGAGMWTLSPPSAWA
>Q07JX7_RHOP5_1 SIGNAL: Potential.
MATNRSRRGRVTLLAVAIAAGPIAFTTADRAR
>Q07P01_RHOP5_1 SIGNAL: Potential.
MLVTHARRNGAPQRSTGWLASLTAIAVAILAYPELSLAQ
>Q0AKS3_MARMM_1 SIGNAL: Potential.
MPHTPTPLTLKSLLVASTAIVGIAAATPAFAQ
>Q0AQ16_MARMM_1 SIGNAL: Potential.
MRRRFLFASALVSLTVTAPAAFAD
>Q0AT43_MARMM_1 SIGNAL: Potential.
MAAVNRGVRTDAKAGAPRRVSMLLAGIALGFSAPVHAA
>Q11GI1_MESSB_1 SIGNAL: Potential.
MNVFETLSKGVSSAAICACIATLPGNLHAQ
>Q11M33_MESSB_1 SIGNAL: Potential.
MKTTAYLKLSGSLIALTAAAVSQAHAQ
>Q12FI0_POLSJ_1 SIGNAL: Potential.
MTLNYSFSYFMQKPIRYSLTAAACLLAFSAQAQ
>Q132Z9_RHOPS_1 SIGNAL: Potential.
MQRMTLAPSRRIAMLWSAALALSASASPALAQ
>Q139I7_RHOPS_1 SIGNAL: Potential.
MGAGRHFRNLSTLFLCTTFLVSAPVSAALYAA
>Q1C198_YERPA_1 SIGNAL: Potential.
MKTNRSTLSPCFRKTMIASLLVPLCSPLYSWAV
>Q1C326_YERPA_1 SIGNAL: Potential.
MNNHKIWRLSAVAVALLISGNSYAD
>Q1C4W8_YERPA_1 SIGNAL: Potential.
MDKTLLAGAISLSLVILPVQVLAF
>Q1C5I0_YERPA_1 SIGNAL: Potential.
MKSRHHLNTRLLPLSILISALIPAAVLAA
>Q1C5I1_YERPA_1 SIGNAL: Potential.
MKNSNTLNTRLLPLSILISSLVSGGAMAV
>Q1CAV8_YERPA_1 SIGNAL: Potential.
MHNKFKANTLAISIAAILLSVSFNTLAV
>Q1CDG9_YERPN_1 SIGNAL: Potential.
MKTNRSTLSPCFRKTMIASLLVPLCSPLYSWAV
>Q1CF77_YERPN_1 SIGNAL: Potential.
MHNKFKANTLAISIAAILLSVSFNTLAV
>Q1CK98_YERPN_1 SIGNAL: Potential.
MKNSNTLNTRLLPLSILISSLVSGGAMAV
>Q1CK99_YERPN_1 SIGNAL: Potential.
MKSRHHLNTRLLPLSILISALIPAAVLAA
>Q1CKV3_YERPN_1 SIGNAL: Potential.
MDKTLLAGAISLSLVILPVQVLAF
>Q1CMH9_YERPN_1 SIGNAL: Potential.
MNNHKIWRLSAVAVALLISGNSYAD
>Q1GU46_SPHAL_1 SIGNAL: Potential.
MRKTLLASTCLATLLSTAVHAE
>Q1MHZ0_RHIL3_1 SIGNAL: Potential.
MRIYRWLSASVGRHVGLATLFAGMALFLDAYG
>Q1MK18_RHIL3_1 SIGNAL: Potential.
MSPFCGSPTVLFSLLIPGTIMGGD
>Q1QCE5_PSYCK_1 SIGNAL: Potential.
MPRNISHAIVNPTTLKTLTKSMLAISLSMAGLAHAE
>Q214Q0_RHOPB_1 SIGNAL: Potential.
MGAGFFRDVSKLLLCTTFLVAAPVSAVLQAA
>Q2GAY1_NOVAD_1 SIGNAL: Potential.
MDRLRTTTILSTLAGTPVALALLVPQAANAA
>Q2J1P8_RHOP2_1 SIGNAL: Potential.
MQKARTRILAGFAFAMATSVSTGAVAAC
>Q2KTY1_BORA1_1 SIGNAL: Potential.
MTQYPARRPPSHALTAVVLALSSLA
>Q2Y8Z0_NITMU_1 SIGNAL: Potential.
MAKRKKSASLSLYAKFIIALLMAPVASLSSRAQ
>Q3BM38_XANC5_1 SIGNAL: Potential.
MSTNCTNMAAGVRVVLRWPLVFALLLLSTLYSGKAAA
>Q3BVT0_XANC5_1 SIGNAL: Potential.
MKKQDVARTVLASALAVALTACG
>Q3K4D0_PSEPF_1 SIGNAL: Potential.
MPIQCKYKLQHLVLAVALAVGCVEFSLAE
>Q3K4D1_PSEPF_1 SIGNAL: Potential.
MPFPPQRLSFAIALLIATSAAHGK
>Q3K5V0_PSEPF_1 SIGNAL: Potential.
MIKQTLFVPLAGCLLAMACAQANAA
>Q3KC67_PSEPF_1 SIGNAL: Potential.
MQRKISNVRLRDIRWGLVLSSFLAPFSQIAIGG
>Q3KCT0_PSEPF_1 SIGNAL: Potential.
MKNNNTPAQSGGGFRLKTLNVALLCAMATWGSAHAA
>Q3KCT1_PSEPF_1 SIGNAL: Potential.
MDVRIKPISVGTLLLVISATQAQAQ
>Q3KD77_PSEPF_1 SIGNAL: Potential.
MKTSLTSEEIKTTFCTVSSSILLCSSMEAQAG
>Q3KDN6_PSEPF_1 SIGNAL: Potential.
MFPRFLCSLSVLSLSIAAVHAA
>Q3SNP6_NITWN_1 SIGNAL: Potential.
MNVVVRASMPGNGALRRRVVAGGAFAFALSASSGAIAA
>Q4ZMI6_PSEU2_1 SIGNAL: Potential.
MTKTSRRWPFAACLLSLACGTAAAA
>Q4ZQ07_PSEU2_1 SIGNAL: Potential.
MQKSKCVGVVRYSFKPVATGALCALSFTFGCSAYAD
>Q4ZUU5_PSEU2_1 SIGNAL: Potential.
MNAPFVLRPLSWTLKTVIFLSPLLPGSHAFAQ
>Q4ZYT2_PSEU2_1 SIGNAL: Potential.
MFRKTLLAMAMAATAVPACAE
>Q664E5_YERPS_1 SIGNAL: Potential.
MKQNRSTLSPCFRKTLIASLLVPLCSPLYSWAV
>Q665R2_YERPS_1 SIGNAL: Potential.
MNNHKIWRLSAVAVALLISGNGYAD
>Q667C1_YERPS_1 SIGNAL: Potential.
MHNKFKANTLAISIAAILLSVSFNTLAV
>Q667Z0_YERPS_1 SIGNAL: Potential.
MKSRHHLNTRLLPLSILISALIPAAVLAA
>Q667Z1_YERPS_1 SIGNAL: Potential.
MKSRSNLNTRLLPLSILISSLIPGAVLAA
>Q667Z2_YERPS_1 SIGNAL: Potential.
MKNSNTLNTRLLPLSILISSLVSGGAMAV
>Q66DI5_YERPS_1 SIGNAL: Potential.
MDKTLLAGAISLSLVTLPVQVLAF
>Q6N1B5_RHOPA_1 SIGNAL: Potential.
MRRAASCQSACLPTVIPLSIAE
>Q6N8G7_RHOPA_1 SIGNAL: Potential.
MSTVGRFRHLSSLLLCTTFLVSAPMSAVLYAA

## Figures and Tables

**Table 1:** NtraC analysis of 90 long bacterial signal peptides.

Nr.	Accession number^1^	SP length^2^	NtraC^3^	N-domain^4^	C-domain	Predicted C-domain targeting	Organism
1	NP_052685	55	yes	1–26	27–55	gram−5	*E.coli*^6^
2	AAC44731/AAG37043	53	yes	1–17(1–35)	18–53(36–53)	Gram−	*E.coli*^7^
3	CAA11507	52	yes	1–17	17–52	gram−	*E.coli*^8^
4	AAD23953	55	yes	1–15(16)	17–55	SP/SA^9^	*E.coli*^10^
5	CAA88252	56	no	–	–	–	*S.flexneri*
6	CAA46156	42	yes	1–26	27(28, 29)–42	gram+	*E.coli*^11^
7	CAC14227	75	yes	1–40	41–75	Gram−	*Y.pestis*
8	AAC43721	50	no	–	–	–	*H.influenzae*
9	CAC14202	62	yes	1–27(–44)	28–62	Gram−	*P.multocida*
10	AAK68872/AAK09243	51	yes	1–26	27–51	gram−	*N.meningitides*
11	AAG01335	66	yes	1–27	28–66	gram−	*X.oryzae*
12	CAD12824/AAA22974	71	yes	1–28(–44)	29–71	gram−	*B.pertussis*
13	CAI77662	70	yes	1–27	28–70	gram−	*H.influenzae*
14	AAM88788	62	yes	1–38	39–62	gram−	*P.luminescens*
15	NP_253231	53	yes	1–24	25–53	mtp, SP, gram+	*P.aeruginosa*
16	NP_252771	52	yes	1–23	24–52	gram−	*P.aeruginosa*
17	AAA20524	68	yes	1–26(mTP); 1–35	35–68	gram−	*H.influenza*
18	AAB96359	48	yes	1–15(1–25)	16–48(26–48)	gram−	*M.catarrhalis*
19	AAF40927	78	yes	1–43(1–50)	44–78(51–78)	gram−	*N.meningitis*
20	AAF67320	54	yes	1–30	31–54	gram−	*S.flexneri*
21	AAG30168	49	yes	1–29	30–49	gram−	*E.coli*^12^
22	AAK00474	52	yes	1–33(1–36) (mTP,SP)	37–52	–	*S.flexneri*
23	AAK77860	69	yes	1–43	44–69	gram−	*Y.enterocolitica*
24	AAL18821	52	yes	1–15(1–29)	16–52(30–52)	gram−	*E.coli*^13^
25	AAL78284	67	yes	1–30(39)	31(40)–67	gram−	*M.catarrhalis*
26	AAQ22366	56	no	–	–	–	*A.actinomycetemcomitans*
27	CAA88252	56	no	–	–	–	*S.flexneri*
28	CAC14203	71	yes	1–26	27–71	Gram−	*P.multicoda*
29	CAC14218	84	yes	1–27 (48)	28(49)–81	SP	*A.ferrooxidans*
30	CAC39286	52	yes	1–16 (29)	17(30)–52	gram−	*E.coli*^14^
31	CAC92482	69	yes	1–37(41)	38(42)–69	gram−	*Y.pestis*
32	NP_274768	80	yes	1–48(49)mTP	49(50)–80	gram−	*N.meningitis*
33	NP_308389	48	yes	1–15(24)(38)	16(25)(39)–48	mTP^15^, SA, SP	*E.coli*^16^
34	NP_519008	72	yes	1–36(37)(39)	33(–40)–72	gram−	*R.solanacearu*
35	NP_519896	60	yes	1–30(–38)	31(–39)–60	gram−	*R.solanacearu*
36	NP_522634	66	yes	1–33(34)(41)	34(35, 42)–66	gram−	*R.solanacearu*
37	NP_636050	66	yes	1–43(55,56,58)	27(28)(31)(44)–66	gram−	*X.campestris*
38	NP_807449	50	yes	1–32(39)	29(39)–50	SP, mTP	*S.enterica*
39	YP_553065	57	yes	1–37(mTP)	30(–38)–57	gram−	*B.xenovarans*
40	ZP_00033562	57	yes	1–28(29)	29(30)–57	gram−	*B.fungorum*
41	ZP_00041732	67	yes	1–58(mTP)	30(33, 35)–67	SP	*X.campestris*
42	ZP_00088699	49	yes	1–32(mTP)	18(29, 32, 33)–49	gram−	*A.vinelandii*
43	ZP_00122019	77	yes	1–40	41(43, 48, 52, 53)–77	gram−	*H.somnus*
44	YP_719000	69	yes	1–37	38(39, 44, 49)–69	SP	*H.somnus*
45	ZP_00132251	69	yes	1–40	41(42, 43)–69	gram−	*H.somnus*
46	AAC26634	52	no	–	–	–	*E.coli*^17^
47	AAC74583	61	yes	1–29(40, mTP)	30(41, 42)–61	gram−	*E.coli*^18^
48	AAD41751	54	yes	1–15(27, 28, 29)	16(28, 29, 30)–54	gram−	*E.coli*^19^
49	AAF43424	52	yes	1–32	33–52	gram−	*E.coli*^20^
50	AAG53941	65	yes	1–38 (mTP)	23(29, 30, 39)–65	gram−	*B.bronchiseptica*
51	AAP33781	52	yes	1–16	17–52	gramp+, SP	*E.coli*^21^
52	Q84GK0	56	yes	1–16	17–56	gram+	*E.coli*^22^
53	Q7BCK4	52	yes	1–16(17)	18–52	gram+, SP	*S.flexneri*
54	Q8CWC7	55	yes	1–16	17–55	SA	*E.coli*^23^
55	YP_840389	41	yes	1–24(SA, mTP)	25–41	SP	*B.cenocepacia*
56	YP_916859	40	yes	1–19(20)	21–40	gram−, SP	*P.denitrificans*
57	YP_001110606	47	yes	1–18(19)	20–47	gram−, SP, gram+	*B.vietnamiensis*
58	YP_001161762	50	yes	1–15(28)	16(32)–50	gram−, SP, gram+	*Y. pestis*
59	YP_001165485	48	no	–	–	–	*Enterobacter sp. 638*
60	YP_001281217	41	no	–	–	–	*Psychrobacter sp. PRwf–1*
61	YP_001371305	40	no	–	–	–	*O.anthropi*
62	YP_001371348	45	yes	1–25(mTP)	26–45	SP, gram−	*O.anthropi*
63	YP_001373319	45	yes	1–20	21–45	gram−, SP, gram+	*O.anthropi*
64	YP_001948780	41	yes	1–18(24)	25–41	gram−, SP, gram+	*B.multivorans*
65	YP_001683300	42	yes	1–13(23)	24–42	gram−, SP, gram+	*Caulobacter sp. K31*
66	YP_001766852	47	yes	1–28(mTP)	29–47	gran negative SP, SP	*M.radiotolerans*
67	ZP_02906872	42	no	–	–	–	*B.ambifaria*
68	YP_001811545	42	no	–	–	–	*B.ambifaria*
69	YP_001990882	50	yes	1–18(–23)	24–50	gram−, SP, gram+	*R.palustris*
70	YP_001993037	45	yes	1–19	20–45	gram−, SP, gram+	*R.palustris*
71	YP_001993508	46	yes	1–19	20–46	gram−, SP, gram+	*R.palustris*
72	YP_002027122	40	no	–	–	–	*S.maltophilia*
73	Q0AL61	45	yes	1–13(29,35)mTP	14(30, 36)–45	gram−, SP, gram+	*M.maris*
74	Q0B7F7	47	yes	1–17(1–33 mTP)	18–47	gram−, SP, gram+	*B.cepacia*
75	Q0PAN9	40	yes	1–17	18–40	gram−, SP, gram+	*C.jejuni*
76	Q1MKD9	40	yes	1–27(29) (mTP)	30–40	–	*R.leguminosarum*
77	Q20XG2	49	yes	1–15(–20)	21–49	gram−, SP, gram+	*R.palustris*
78	Q214C9	50	yes	1–19	20–50	gram−, SP, gram+, mTP	*R.palustris*
79	Q216F9	42	yes	1–14(–18)	19–42	gram−, SP, gram+	*R.palustris*
80	Q2J0N4	44	(yes)	1–37(mTP)	38–44	–	*R.palustris*
81	Q48237	48	yes	1–25	26–48	gram−, SP, gram+	*H.mustelae*
82	CAR56027	41	(yes)	1–30(31)(SA)	32–40	–	*B.cenocepacia*
83	Q132B9	44	yes	1–17	18–44	gram−, SP, gram+	*R.palustris*
84	Q137J7	43	yes	1–26(27)	28–43	gram−, SP	*R.palustris*
85	Q2KVL9	47	yes	1–15	16–47	gram negatice SP	*B.avium*
86	Q4ZQ08	43	yes	1–29(mTP)	30–43	SP	*P.syringae*
87	Q6N2N9	46	yes	1–19	20–46	gram−, SP, gram+	*R.palustris*
88	Q6N3Z4	46	yes	1–17(–21)	22–46	Gram−, SP, gram+	*R.palustris*
89	Q6N964	46	yes	1–18(23)	24–46	gram−, SP, gram+	*R.palustris*
90	P39180	52	yes	1–35(u.–29 mTP)	36–52	SP, gram+ (30–52)	*E.coli*^24^

1 Nr. 1–16 taken from Szabady et al.^10^; Nr. 17–51 taken from Henderson et al^10^; Nr. 52–90 retrieved via SRS.

2 gives the position of the last residue of the signal peptides as used in the publications or annotated in UniProtKB v. 14.7.

3 NtraC organization of the sequence.

4 Length of the predicted N- or C-domain. Numbers in brackets refer to alternative possibilities for truncation of the domains. A targeting abbreviation in brackets means: only that length combination leads to the targeting function in brackets. **Predicted C–domain targeting:** predicted by SignalP or TargetP.

5 −/+: gram negative or gram positive signal peptide.

6 strain = Sakai.

7 strain = E2348/69.

8 strain = EB1.

9 SP: eukaryotic signal peptide, SA: signal anchor gram.

10 strain = 042.

11 strain = K–12.

12 strain = CFT073.

13 strain = EH41.

14 strain = 4797/97.

15 mitochondrial targeting peptide.

16 strain = Sakai.

17 strain = 042.

18 strain = K–12.

19 strain = H10407.

20 strain = ML308–225.

21 strain = APEC13.

22 strain = O78:H11.

23 strain = O6:H1.

24 strain = K12.

**Table 2:** Mean occurrence of charged residues given in one letter code in 79 NtraC-organized autotransporter signal sequences the length of the transition area (tra) is up to eight residues.

	His	Lys	Arg	Asp	Glu
N-domain^25^ (−*tra*)^26^	0.5 (±0.8)^27^	1.9 (±1.5)	2.3 (±1.7)	0.4 (±0.6)	0.8 (±0.7)
C-domain (+*tra*)	0.3 (±0.5)	1.1 (±1.4)	1.5 (±1.4)	0.2 (±04)	0.3 (±0.6)
N-domain (+*tra*)	0.6 (±0.8)	2.3 (±1.8)	2.6 (±1.8)	0.4 (±0.7)	0.9 (±0.7)
C-domain (−*tra*)	0.2 (±0.5)	0.8 (±1.0)	1.1 (±1.2)	0.1 (±0.4)	0.2 (±0.5)

25 Domains of the NtraC algorithm.^8^

26 Excluding or including the transition area (domain border) predicted by the NtraC model.

27 Standard deviation in brackets.
